# Geminivirus data warehouse: a database enriched with machine learning approaches

**DOI:** 10.1186/s12859-017-1646-4

**Published:** 2017-05-05

**Authors:** Jose Cleydson F. Silva, Thales F. M. Carvalho, Marcos F. Basso, Michihito Deguchi, Welison A. Pereira, Roberto R. Sobrinho, Pedro M. P. Vidigal, Otávio J. B. Brustolini, Fabyano F. Silva, Maximiller Dal-Bianco, Renildes L. F. Fontes, Anésia A. Santos, Francisco Murilo Zerbini, Fabio R. Cerqueira, Elizabeth P. B. Fontes

**Affiliations:** 1 Departamento de Informática, Universidade Federal de Viçosa, Viçosa, Brazil; 2 National Institute of Science and Technology in Plant-Pest Interactions/BIOAGRO, Universidade Federal de Viçosa, Viçosa, Brazil; 3 Núcleo de Biomoléculas, Universidade Federal de Viçosa, Viçosa, MG Brazil; 4 Departamento de Bioquímica e Biologia Molecular, Universidade Federal de Viçosa, Viçosa, Brazil; 5 Departamento de Zootecnia, Universidade Federal de Viçosa, Viçosa, Brazil; 6 Departamento de Solos, Universidade Federal de Viçosa, Viçosa, Brazil; 7 Departamento de Biologia Geral, Universidade Federal de Viçosa, Viçosa, Brazil; 8 Departamento de Fitopatologia, Universidade Federal de Viçosa, Viçosa, MG Brazil; 9 Departamento de Engenharia de Produção, Universidade Federal Fluminense, Petrópolis, Rio de Janeiro Brazil

**Keywords:** Machine learning, Random Forest, Knowledge discovery, Data mining, Data Warehouse, Geminivirus

## Abstract

**Background:**

The *Geminiviridae* family encompasses a group of single-stranded DNA viruses with twinned and quasi-isometric virions, which infect a wide range of dicotyledonous and monocotyledonous plants and are responsible for significant economic losses worldwide. Geminiviruses are divided into nine genera, according to their insect vector, host range, genome organization, and phylogeny reconstruction. Using rolling-circle amplification approaches along with high-throughput sequencing technologies, thousands of full-length geminivirus and satellite genome sequences were amplified and have become available in public databases. As a consequence, many important challenges have emerged, namely, how to classify, store, and analyze massive datasets as well as how to extract information or new knowledge. Data mining approaches, mainly supported by machine learning (ML) techniques, are a natural means for high-throughput data analysis in the context of genomics, transcriptomics, proteomics, and metabolomics.

**Results:**

Here, we describe the development of a data warehouse enriched with ML approaches, designated geminivirus.org. We implemented search modules, bioinformatics tools, and ML methods to retrieve high precision information, demarcate species, and create classifiers for genera and open reading frames (ORFs) of geminivirus genomes.

**Conclusions:**

The use of data mining techniques such as ETL (Extract, Transform, Load) to feed our database, as well as algorithms based on machine learning for knowledge extraction, allowed us to obtain a database with quality data and suitable tools for bioinformatics analysis. The Geminivirus Data Warehouse (geminivirus.org) offers a simple and user-friendly environment for information retrieval and knowledge discovery related to geminiviruses.

**Electronic supplementary material:**

The online version of this article (doi:10.1186/s12859-017-1646-4) contains supplementary material, which is available to authorized users.

## Background

The advancement of high-throughput sequencing technologies has enabled the rapid increase of genomic data in public databases and introduced genomics into the era of massive data generation. The biggest challenges, thus, turned out to be how to acquire, classify, store, and analyze huge datasets and extract knowledge from them [1]. Furthermore, the processing of massive data analysis has additional challenges, such as how to feasibly address bulky data, how to speed up the processing, and how to maintain the data veracity.

To extract and process data of interest, it is recommended to use the process known as Knowledge Discovery in Databases (KDD) process by which the data are selected, preprocessed, transformed, mined, and evaluated [2, 3]. The data mining step includes the application of unsupervised and supervised methods such as clustering analysis, classification, and rule learning techniques [4]. Machine learning (ML) techniques and data mining applications have been suggested for high-throughput data analysis in plants as well for all levels of studies, i.e., in genomics, transcriptomics, proteomics, and metabolomics [5], including taxonomic classification in metagenomic data [6]. The current high-throughput sequencing methods, metagenomics analysis approaches, and powerful bioinformatics tools accelerated knowledge acquisition of a number of viromes, allowing the identification of several viral agents in a wide range of cultivated and uncultivated plants. Furthermore, using rolling-circle amplification approaches, thousands of full-length geminivirus and satellite genome sequences have been amplified and have become available in public databases [7–9] (www.ictvonline.org).

The *Geminiviridae* family is a group of single-stranded DNA (ssDNA) viruses, with twinned and quasi-isometric virions, which infects a wide range of dicotyledonous and monocotyledonous plants and is responsible for important economic losses in tropical and subtropical regions worldwide. The *Geminiviridae* family is composed of nine genera: *Becurtovirus*, *Begomovirus*, *Curtovirus*, *Eragrovirus*, *Mastrevirus*, *Topocuvirus*, *Turncurtovirus, Capulavirus* and *Grablovirus* [10, 11]. The current classification is based on their insect vector, host range, genome organization, and phylogeny reconstruction [7, 8]. Except for viruses in the genus *Begomovirus,* which can be monopartite (single genomic DNA) or bipartite (two DNA components, referred to as DNA-A and DNA-B), all geminiviruses from the other genera have a single genomic component. The DNA-A of begomoviruses contains genes required for DNA replication (*Rep*, *REn*), gene expression control (*TrAP*), suppression of host defenses (*TrAP* and *AC4*), and viral genome encapsidation (*CP*), whereas the DNA-B encodes two proteins involved in intra- and intercellular movement (NSP and MP) [9, 12]. The single genomic component of mastreviruses encodes four proteins: a movement protein (pre-coat), a coat protein (CP), and two splicing variants of the replication-associated protein (Rep) [13]. The genomic structure of becurtoviruses contains five genes: the pre-coat gene, a *CP*, two *Rep*s, and possibly a regulatory gene (*Reg*) [14, 15]. Viruses from the genera *Eragrovirus* and *Turncurtovirus* encode a pre-coat protein, CP, Rep, and transactivator protein (TrAP). However, turncurtoviruses encodes two additional proteins, the replication enhancer (Ren) and Symptom determinant/possible symptom determinant proteins (Sd/p.sd) [7, 16–18]. The genomic structure of curtoviruses is composed of seven genes, including the pre-coat gene, *Reg*, *CP*, *Ren*, *TrAP*, *Rep*, and *Sd/p.sd* [19, 20]. The genus *Topocuvirus* has only one genome sequence deposited in public databases, which is organized into six genes, a pre-coat gene, *CP*, *Ren*, *TrAP*, *Rep*, and *Sd/p.sd* [21]. The recently discovered *Capulavirus* and *Grablovirus* genera encompass viruses that share similar genomic organization with becurtoviruses and eragroviruses [11].

Typically, the “Old World Geminiviruses” (from Europe, Asia, and Africa) are predominantly monopartite and commonly associated with alpha- or betasatellite DNAs, whereas “New World Geminiviruses” (from the Americas) are predominantly bipartite and may be associated with alphasatellites [22]. The betasatellite genome is approximately 1.35-kb long and harbors a single Open Reading Frame (ORF), *β*C1 [23]. The alphasatellite genome is approximately 1.37-kb long and contains a single ORF, which encodes a rolling-circle replication-initiator protein (*Rep*) [24, 25].

Unlike other important viral pathogens, such as Hepatitis C (hcv.lanl.gov) and HIV (hiv.lanl.gov), no database has been developed, which integrates all relevant information and provides user-friendly tools enriched by ML approaches for the easy manipulation of geminivirus data. Large amounts of information are distributed in a wide range of databases and in different file formats (for example GenBank, UniProt, VIPR, and ViralZone). Acquiring access to this information is usually a complex and time-consuming task. Additionally, a high level of computational expertise is required. To overcome these limitations, we developed a new data warehouse, designated Geminivirus Warehouse (geminivirus.org), using the concepts of the KDD process. The data warehouse geminivirus.org uses the ETL (Extract, Transform, Load) process, commonly applied for data warehouses, to choose, curate sequences, and standardize data. The geminivirus.org data warehouse is enriched with ML methods for the classification of the viral genus using the genomic sequence and the identification of gene coding sequences. The computational tools also comprise species demarcation, and include advanced bioinformatics tools for basic local alignment search, pairwise sequence comparison, including construction of the respective identity matrix, and phylogenetic analysis. Furthermore, we developed an algorithm for the ORF prediction from the genomes of each genus with high accuracy and which is capable of identifying possible intron regions.

## Construction and content

### Implementation

**Fig. 1:**
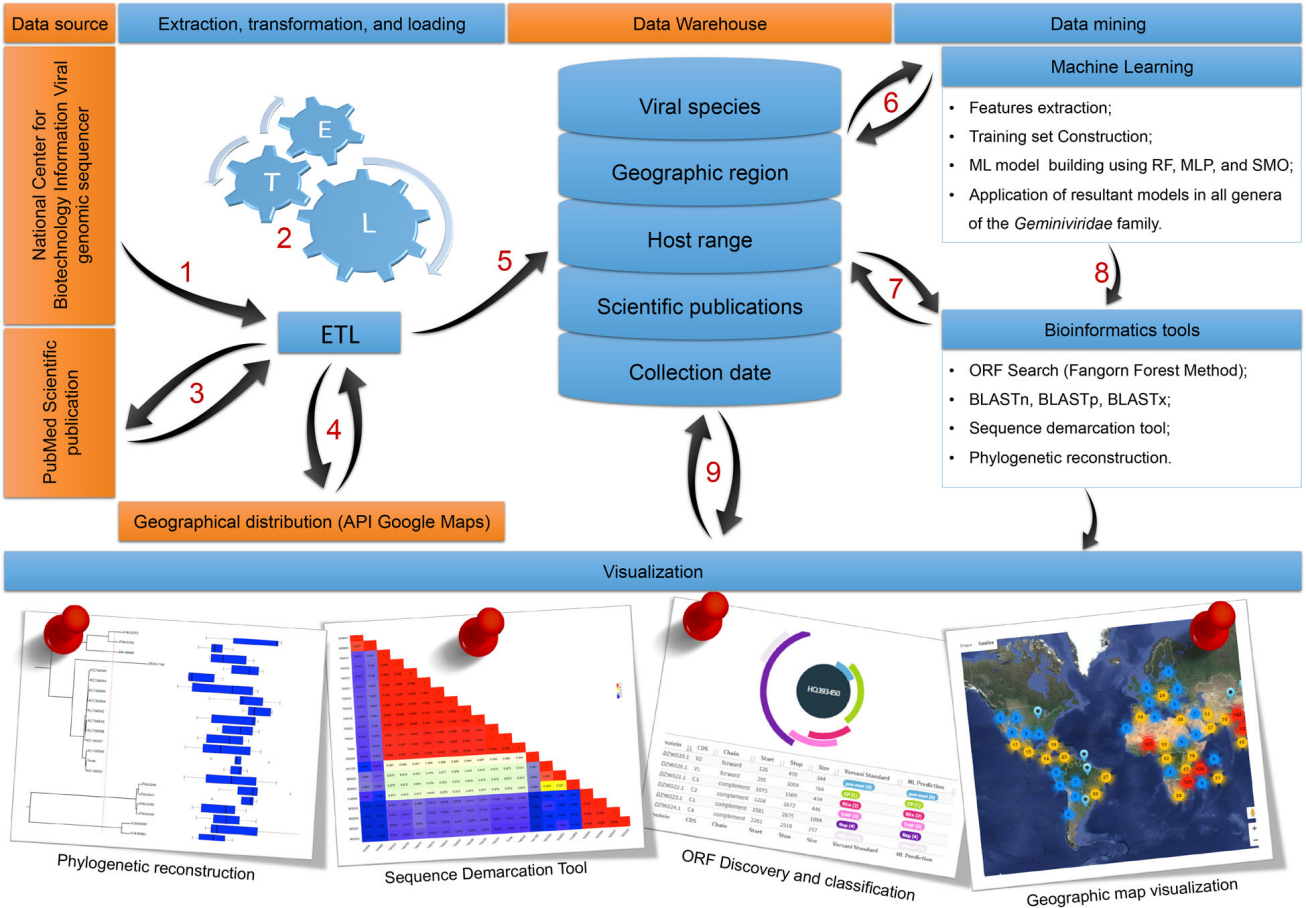
Overview of the geminivirus.org framework. Initially, the geminivirus data were recovered from GenBank in the GenBank file format (*1*). The data were extracted, transformed, and standardized using algorithms based on rules and machine learning (ML) approaches (*2*). Next, the abstracts of the scientific publications were recovered from PubMed (https://www.ncbi.nlm.nih.gov/pubmed) (*3*) and the geographic coordinates of the isolates were retrieved from Google Maps (*4*). Data were merged and loaded into the relational database (*5*) in different dimensions such as the collection date, host range, geographic region, genomic data, associated publications, and organism data. The data were used to define the training set for building ML models to classify genera using Random Forest (RF), Multilayer Perceptron (MLP), and Sequential Minimal Optimization (SMO) learning algorithms (*6*). Information and analytical tools, such as basic local alignment tools (BLAST), sequence demarcation tools, and phylogenetic reconstruction, were embedded in the system (*7*) and an ORF Search tool for classification of ORFs based on ML procedures (*8*) was implemented. All analysis results are visible and freely available (*9*)

The geminivirus.org data warehouse was implemented using an MVC (Model-View-Controller) software architecture pattern with modules programmed in the Java programming language and with MYSQL server relational databases. The data warehouse structure was organized in SQL tables in the star format [26] and geminivirus.org was developed using the KDD concepts, in which the ETL process was applied and ML algorithms from the Weka library v3.7.11 [27] were used. The design and workflow are summarized in Fig. 1 and detailed in the following sections.

### Data source

Initially, geminivirus-related data were obtained in a document format from the GenBank nucleotide database. This file format contains information related to the complete genome, country of origin, geographic coordinates of the region or province, collection date, host, author responsible for the collection, among others. Notably, all of these data are not always present, and the document structure is often out of the standard. Next, the article abstracts relating to the retrieved sequences were retrieved from the PubMed database. Finally, the geographic information such as the country name, geographic region or geographical coordinates obtained from GenBank were used to retrieve the geographical coordinates in the UTM (Universal Transverse Mercator) format of Google Maps.

### Raw data extraction

Preliminarily, information was extracted from the GenBank file, as described in Table 1. Then, each file record was inspected using the following criteria concerning the full-length genome sequences: i) the length must be greater than or equal to the minimum predefined size, ii) the length cannot exceed the maximum length, and iii) they must fit one of the nine genera of the *Geminiviridae* family (Additional file 1: Table S1). Genomes of alpha- and betasatellites were also included, as well as unassigned genomes that have not yet been classified by ICTV (https://talk.ictvonline.org/taxonomy). Thereafter, each pre-selected record was stored as a candidate to join the data warehouse sequences.

**Table 1:** Example of information extracted from the GenBank file and stored in geminivirus.org

TAGs	Value
LOCUS	KJ939916
DEFINITION	Soybean chlorotic spot virus isolate BR:Flt14:11 segment DNA-A, complete sequence.
ORGANISM	Soybean chlorotic spot virus
PUBMED	25028472
AUTHORS	Sobrinho,R.R., Xavier,C.A.D., Pereira,H.M.B., Lima,G.S.A.,,Assuncao,I.P., Mizubuti,E.S.G., Duffy,S. and Zerbini,F.M.
.,JOURNAL Submitted	Departamento de Fitopatologia, BIOAGRO, Universidade Federal de Vicosa, Av. Peter Henry Rolfs s/n, Vicosa, Minas Gerais 36570–900, Brazil
Assembly Method	CodonCode Aligner v. 4.1.1 DEMO
Sequencing Technology	Sanger dideoxy sequencing
host	Macroptilium lathyroides
taxon	1221206
country	Brazil
segment	DNA-A
lat_lon	
collection_date	18-Mar-2012
collected_by	
CDS	199..954
gene	
note	coat protein
product	CP
protein_id	AIN36521.1
translation	MVKRDAPWRHMAGTSKVSRSSNFSPRGGGGPKNNRTSEWVNRPM …
ORIGIN	ACCGGATGGCCGCGCGATTTTTTATGGGCCTTATCTTTTGGCTCGTTCTTTTGGACCGAGTGTATTTGAATTAAAGTAAAGTTATTCCCTGTCCAA................

### Data transformation

After the extraction step, it is necessary to transform and standardize the data, as well as to correct errors and relate different information or data from heterogeneous sources to improve the quality and consolidate the data [28]. In addition, it is necessary to associate metadata to data of interest entered into the database [29]. To perform these steps, the pre-selected records were processed using the following criteria incorporated in Algorithm 1:

(i) **Origin of replication.** Firstly, corrections were performed in genome sequences that do not start at the expected genomic coordinates. These genome regions were adjusted to start in the first nucleotide after the cleavage site (dash) within the conserved nonanucleotide at the geminivirus replication origin (TAATATT-AC) and geminivirus-associated alpha- and betasatellite DNAs;

(ii) **Repairing the Open Read Frame coordinates in the genome.** The start and stop codon coordinates of each gene belonging to the genome sequence adjusted in step i were redefined according to the adjustment performed;

(iii) **Genus classifier in the** ***Geminiviridae*** **family.** The genera were confirmed using ML approaches;

(iv) **Checking the consistency of ORFs.** We verified whether all coding DNA sequences had start and stop codons, and whether the amino acid sequences were not truncated;

(v) **Standardization of gene acronyms.** The standardization of acronyms for gene identification was conducted following the genomic organization of the nine genera of the *Geminiviridae* family (Table 2). The following acronyms were used: CP, capsid protein; Rep, replication-associated protein; TrAP, transactivator protein; Ren, replication enhancer; MP, movement protein; NSP, nuclear shuttle protein; Reg, regulatory gene; Sd, symptom determinant; Ss, silencing suppressor; Tgs, transcriptional gene silencing. Note that the DNA-A component of old-world bipartite begomoviruses contains a V2 ORF, defined as a pre-coat in our standardization [8];

**Table 2:** Terms used to name CDS in NCBI

Genera	CDS term NCBI	Varsani standard
Betasatellite	“beta” or “c1”	betaC1
Alphasatellite	“alpha” or “rep”	alphaRep
Begomovirus	“bv1” or “nsp” or “nuclear shuttle”	NSP
Begomovirus	“bc1” or “bc2” or “mp”	MP
All genera	“c1” or “ac1” or “rep” or al1	Rep
All genera	“c2” or “ac2” or “trap” or “al2” or “transcription activator protein”	TrAP
All genera	“c3” or “ac3” or “ren” or al3	REn
All genera	“c4” or “ac4” or al4	sd/p.sd
All genera	“c5” or “ac5”	AC5
All genera	“v1” or “av1” or “cp” or “ar1” or “capsid protein” or “coat protein”	CP
All genera	“v2” or “av2” or “pre-coat” or “precoat” or ar2	pre-coat
All genera	“v3” or “av3”	Reg

(vi) **ORF classifier in each genus of the** ***Geminiviridae*** **family.** In this step, we confirmed whether the ORFs were correctly standardized using ML approaches;

(vii) **Standardization of country abbreviation.** Country and continent abbreviations for all genera were standardized;

(viii) **Standardization of species name.** The species names were replaced following a list of begomovirus species, as of January 2015 [9], available at the ICTV website (https://talk.ictvonline.org/ictv_wikis/geminiviridae/m/files_gemini/5120/download);

(ix) **Recovering geographic coordinates.** We recovered geographical coordinates with exact (deposited coordinates) or approximate positions through secondary information, such as the informed country;

(x) **Recovering scientific publications.** All scientific publications related to a deposited sequence were recovered.

Our programs were developed using object-oriented programming concepts. We implemented a collection of classes designated as Object Geminivirus (OG). The instances of the OG classes have the purpose of storing information, performing tasks (e.g., create, read, update, delete data in database), and communicating between the application (user interface) and the database. OG objects are instantiated with the data just after their transformation, preparing them to be loaded into our data warehouse.

### Data load

The storage structure of our data warehouse was modeled in SQL tables with an adapted star scheme (Additional file 1: Figure S1). The star scheme is composed of one or more fact tables that represent data as facts. For instance, each isolate or organism can have these facts: (i) genome sequence and open reading frames, (ii) geographical localization, (iii) collection date, (iv) host range, (v) authors and related institutions and (vi) scientific publication reference. To insert the data into SQL tables, the transformed data were loaded to the OG object, by which each full-length genome and its associated metadata were inserted into the database, maintaining the integrity of data in different star scheme tables. It is worth mentioning that the OG object allows for control of all changes and transformations performed in a sequence and their associated metadata to record the history of changes and additional information. Other information regarding genome sequences which was not available on the GenBank Database was manually updated by our team, who inspected several scientific articles and afterwards inserted the information into the data warehouse.

### Data mining

#### Machine learning

##### Datasets

As mentioned above in steps iii and vi of data transformation, the genome sequences and complete ORFs are classified using ML approaches. For genus classification, complete genomes of species from the nine genera as well as satellite genome sequences were used to create the training set instances. As a result, the possible class labels are *Begomovirus*, *Mastrevirus*, *Curtovirus*, *Becurtovirus*, *Eragrovirus*, *Turncurtovirus*, *Topocuvirus*, *Capulavirus*, *Grablovirus*, alphasatellite, and betasatellite. The genus genomes were selected according to taxonomic reviews [7, 9, 11, 12, 19]. The betasatellite sequences, in turn, were chosen using the study of Briddon et al. [30], while alphasatellites were randomly selected from our curated repository. In addition, a test set was created using sequences contained in geminivirus.org, which were not present in the training set. For ORF classification, the training set was built using the ORFs pertaining to the genome sequences with which the genus training set was constructed. In this case, the instance labels are betaC1, alphaRep, Rep, TrAP, REn, Sd/p.sd, AC5, CP, pre-coat, Reg, MP, and NSP. A test set was also created with ORFs contained in the genomic sequences used for the genus test set mentioned above. The number of sequences, in each class, used to compose the training/test sets for genus and ORF classification, is shown in Additional file 1: Table S2.

##### Classification attributes

In the case of genus classification, each genome sequence, selected to produce an instance in the training or test set, is split into four pieces of same (or nearly same) size, and the following attributes are then collected: proportions of A, T, C, and G of the whole sequence; and proportion of A, T, C, and G as well as GC content of each of the four pieces, totaling 24 attributes.

For ORF classification, attributes were extracted from every coding DNA sequence (CDS) and respective amino acid sequence to produce each instance. In this case, the attributes are proportions of A, T, C, and G of the CDS; and the proportion of each one of the 20 amino acids in the translated CDS.

##### Machine learning algorithm selection

The ML algorithms Sequential Minimal Optimization (SMO), Random Forest (RF), and Multilayer Perceptron (MLP) are easily adaptable to handle multiclass classification problems [31–33], and are largely applied in several recent solutions for bioinformatics problems [34–37]. For this reason, we decided to perform some experiments with these ML approaches to select the best one to be incorporated in geminivirus.org. These algorithms were trained with the training sets presented previously, by means of the WEKA API (v3.7) [27], using the default parameters. The generalization of the resultant models was evaluated using three different techniques:

(i). The use of a completely independent test set;
(ii). 10-fold cross-validation [38]; and
(iii). leave-one-out, which is an *n*-fold cross validation, where *n* is the number of training instances.

To evaluate the performance of classification models, we used the statistical metrics accuracy, precision, recall, and F-measure (equations presented in Additional file 1: Equations S1) as well as the area under the ROC curve (AUC) [39].

Accessing the predictive power of the three models built from the SMO, RP, and MLP approaches for genus classification, MLP and RF presented similar results, with a slight superiority of MLP. Both performed better than SMO. MLP presented the following mean values for accuracy, precision, recall, and AUC, respectively: 0.966, 0.974, 0.967, and 0.986. See detailed results of all tests in Additional file 1:Table S3.

In the same way, the three above-mentioned ML algorithms were tested for ORF classification using the same three evaluation methods. This time, the three resultant models presented a similar predictive power, with a slight superiority of RF over MLP and SMO. RF could achieve mean values for accuracy, precision, recall, and AUC of 0.975, 0.976, 0.976, 0.991, respectively (detailed results in Additional file 1: Table S4). Consequently, geminivirus.org applies the MLP model for genus classification, and the RF model for ORF classification.

### Bioinformatics tools

The data warehouse geminivirus.org provides a user-friendly web interface for the easy usage of advanced bioinformatics tools to search for viral information and to perform basic local alignment search, species demarcation, optimized phylogenetic analysis, ORF discovery and classification, as well as geographical visualization of geminiviruses and satellite-related data:

(i). **User-friendly search modules.** The web interface contains user-friendly search modules for viral sequences and scientific publications. The user can perform a search using keywords, such as viral name, host plant, GenBank Database accession number, country of origin, genome segment (DNA-A, DNA-B, monopartite genome or alpha- and betasatellite), collection year and sequence submission year. The search for scientific publications can also be performed using keywords such as PubMed ID, author name, virus name, scientific journal, and sequence publication year;
(ii). **Basic local alignment search.** To perform a basic local alignment search with sequences of genomes, amino acids, or CDS, we embedded the BLAST software [40] (BLASTn, BLASTp, and BLASTx algorithms) in our platform with pre-adjusted p-value parameters.
(iii). **Species demarcation.** We also incorporated the SDT v1.0 software [41] into geminivirus.org, which enables pairwise-sequence comparison analyses. Query sequences are used for pairwise alignments using MAFFT [42], MUSCLE [43], or ClustalW [44] algorithms. Based on the percentage of sequence identities, desired sequences can be selected to generate a comparative identity matrix. Thus, this matrix can be viewed in geminivirus.org or downloaded to the user’s computer, opened with the original SDT software, and can be edited using any image editing software.
(iv). **Phylogenetic reconstruction analysis.** An automated phylogenetic analysis may be performed in geminivirus.org. The user initially enters at least one query sequence and then performs a search for sequence homology using BLAST algorithms. Query sequences are used to perform pairwise alignments using MAFFT, MUSCLE, or ClustalW algorithms, and the alignment output is automatically loaded into the FastTree software [45]. The phylogenetic analysis is performed using the maximum-likelihood method with 1000 bootstrap replications and other default parameters. The FastTree 2 software uses minimum-evolution subtree-pruning-regrafting and maximum-likelihood NNIs (nearest-neighbor interchange) to search for better trees. We also embedded the Phytools R package into our platform for visualization and additional analysis, for which the fastBM simulation function is used [46]. In addition, the phylogenetic tree output can also be downloaded in the Newick format to the user’s computer, then opened and edited using, for example, the FigTree v1.4.2 software (http://tree.bio.ed.ac.uk/software/figtree).
(v). **Data Visualization.** All information related to geminiviruses and geminivirus-associated satellites, such as viral species and geographical distribution, can be visualized using a graphic interface developed in the Google Maps API (https://developers.google.com/maps/?hl=en) and Google MarkerClusterer (https://github.com/googlemaps/js-marker-clusterer). Additionally, statistical information about the amount of full-length genome sequences per country, viral species, year, and related scientific publications are also shown in charts using the Google Charts API (https://developers.google.com/chart/?hl=en).
(vi). **Discovery and classification of ORFs**. We have developed an algorithm for prediction and classification of genes. Moreover, the algorithm allows the classification of the viral genus based on the genomic sequence using ML approaches.

## Utility and discussion

Geminiviruses infect a wide range of dicotyledonous and monocotyledonous plants causing expensive losses worldwide. A wide range of studies have been published in the literature using genomic data and different bioinformatics, such as studies of molecular interaction mechanisms among viral and host plants [47, 48], population biology [49], species taxonomy [8, 9, 50], and discovery of new viral species by analysis of genetic diversity [51]. In spite of the geminivirus relevance, inflicting serious threat to agriculture in tropical and subtropical areas, there are no databases integrating all relevant related information and providing user-friendly tools for easily manipulating the data. The lack of comprehensive bioinformatics tools for geminivirus analyses motivated the development of a specific database for geminivirus, including automated pipelines to boost findings and the exchange of information among researchers.

The high diversity and amount of viral species complicate the recovery and interpretation of viral genomic and proteomic data. After the advent of the rolling-circle amplification (RCA), using the phi-29 DNA polymerase along with current high-throughput sequencing methods, thousands of full-length sequences have become available from public databases in the last 10 years. This large amount of data is available in a wide range of databases or as supplemental material in scientific publications, such as the full-length genome, coding DNA sequence, geographical localization, host range, data collection, species names, and species identifiers (by acronym). All of these data have great potential to result in new knowledge when unified. Approximately 274 full-length genomic sequences of geminiviruses became available in GenBank databases from 1990 to 2003. Nonetheless, this number has increased exponentially (approximately 34 times) up to the current date (9255 full-length sequences). In parallel, a significant number of scientific papers involving geminiviruses have been reported during the same period.

The number of full-length genomic sequences is distributed among the nine genera and other quantitative information can be found in the data warehouse (http://geminivirus.org:8080/geminivirusdw/statistics.jsp). Furthermore, recently discovered geminiviruses showed that the genetic diversity among genera reaches high levels and, in some cases, presents specific genome architectures [8]. Considering this highly divergent genomic content, we have built a web platform that includes associated metadata, search modules, bioinformatics tools, and ML methods, which retrieve information of interest, demarcate species, and classify genera and ORFs. The following sections provide detailed information on the use of geminivirus.org to retrieve or discover information about geminiviruses.

### Sequence search and data visualization

The search for geminivírus, DNA satellite and gene sequences into Geminivirus Data Warehouse is available through the menu designated Virus. The search and analysis tools provide various searching criteria on both nucleotide sequences (full-length genomes or genes) and amino acid sequences (proteins). The metadata provides the users with the ability to perform searches based on parameters (or combinations thereof), such as viral name, host plant, access number into GenBank Database, country of origin, or genome segment (DNA-A, DNA-B begomoviruses components, alpha- or betasatellite DNAs). After the search, the results are shown in a table format, in which the columns refer to the accession number, sequence description, collection date, submission date in GenBank, host, country, and sequence length. The resultant accession number links all information related to the complete sequence (http://geminivirus.org:8080/geminivirusdw/viewOrgServlet?id=KC706589). In addition to the metadata, the information about the sequence authorship, the funding institutions and those responsible for the data collection (when available in GenBank) was preserved. Information concerning authorship is accessible from the abstract of publications linked to the complete sequences. Furthermore, other detailed information about the consistency and quality of data is presented:

(i). **About ORFs.** Relevant information, such as gene names, virion-sense or complementary strain of the genome, protein sequence and coding DNA sequence are also presented. In addition, the quality of these sequences (presence of start codon, stop codon, and truncation) is inspected and accepted with the appearance of the light blue star and a notice indicating the status of the algorithm verification. However, neglected annotation of submitted sequences is quite common. To overcome this problem, ORFs were classified using learning models constructed with the Random Forest algorithm, as previously mentioned. Thus, the result of the classification is presented to the user, indicating the gene name and the resulting likelihood of classification. This way, the consistency of the ORF and its annotation is reinforced.
(ii). **View of the genome architecture.** The genomic architecture can be viewed in an interactive circular diagram. Furthermore, the genes are shown in a table comprising summary information.
(iii). **Revisions.** During the process of cleaning and processing the data, some changes are performed in the complete genome, CDS, or protein, whereas metadata is included in other data sources or manually entered. The added and changed information is stored in a database, to register the change history. In addition, the history is visible to the users in a change timeline.

The treatments and aggregated information of the submitted sequences are important and positively assist in conducting several studies, such as migration studies, phylogeography analysis, recombination analysis, genetic diversity, and species demarcation, among others. The associated metadata is rated by intuitive icons that represent the sequence quality and reliability. For example, the viral sequences approved in the initial filter receive a yellow medal. On the other hand, sequences associated with at least one publication receive a green medal, and sequences that are inspected and corrected manually receive a red medal. In addition, those sequences confirmed by the Random Forest learning model receive a blue medal. Stars are also used and refer to the existence of a particular metadata. An empty star denotes that the associated metadata is in the process of manual inspection.

### Searching publications

The search for scientific publications can also be performed using keywords, such as PubMed ID, author name, virus name, scientific journal name, and sequence publication year. The search results are presented in a table containing titles, authors, publication year, and PMID number. Clicking on the provided link to each publication enables the access to its abstract, along with other information such as the scientific journal and accession number of the associated virus sequence.

### Basic local alignment search tools

A basic local alignment search can be performed against a query sequence (nucleotide or amino acid) using the BLASTn, BLASTx, BLASTp algorithms embedded in our system. BLAST serves as a tool for searching sequences with higher similarity. The alignment results can be chosen to be automatically used as input in the SDT v1.0 software for species demarcation and also in FastTree for phylogenetic analysis, both embedded in the data warehouse. The BLAST results are merged with other associated metadata, including sequence match, collection date, host, and geographic region. Thus, the tabulated results may help researchers in making decision based on sequence comparisons, host range, and geographical location.

### Species demarcation

A SDT (Sequence Demarcation Tool) was recently implemented for viral species demarcation which provides standardization for all parameters, such as alignments and processing gaps, to calculate the percentage of sequence identity between genomes or gene sequences [41]. We incorporated an adapted parallel version of the SDT software into geminivirus.org (http://geminivirus.org:8080/geminivirusdw/SDT_demarcation.jsp). This enables genome sequences of geminiviruses and associated satellite DNAs to be directly compared and eliminates the need for a local installation of the SDT desktop version in the user’s computer. Briefly, the analysis performs a preliminary comparison of a query sequence to other available sequences in geminivirus.org using BLAST algorithms, which enables a pre-selection of closely related sequences. Then, SDT performs all of the comparisons between the query sequences provided by the user and those sequences that were pre-selected in the previous step of the BLAST results.

Another advantage of using SDT from geminivirus.org is that the algorithm only performs comparisons involving query sequences provided by the user against those available in geminivirus.org, which are the subject sequences of interest. It reduces the analysis complexity and duration needed to generate results. It is important to highlight that the implementation of the SDT program into geminivirus.org in our data warehouse enables the usage of this software from various platforms. Finally, a color array can be obtained, representing the identity percentage values, and can be downloaded as a list containing the results of all pairwise comparisons.

### Phylogenetic reconstruction

The phylogenetic analysis from geminivirus.org enables a rapid visualization of phylogenetic relationships and groupings from the input sequence dataset (http://geminivirus.org:8080/geminivirusdw/phylogeny.jsp). Initially, the sequence of interest is submitted against the geminivirus sequences using the BLASTn algorithm. The selected sequences from the BLAST results are then automatically given as inputs to the MUSCLE algorithm to perform multiple sequence alignments. Next the MUSCLE output is automatically loaded into FastTree. The FastTree is a tool that enables phylogeny inference for alignments with up to hundreds of sequences. It is slightly more accurate than its former version and 100–1000-fold faster than other tools.

### Prediction and classification of ORFs in full-length genome sequences

Collectively, geminiviruses contain ten different known genes. In addition, the alphasatellites encode alphaRep, while betasatellites encode betaC1. The most common way to identify such genes is through the ORF finder tool (www.ncbi.nlm.nih.gov/projects/gorf/). However, prediction and *in silico* annotation of these ORFs require computational expertise and time to process and analyze the data. To address this restriction, we developed a method of prediction and classification of ORFs designated the Fangorn Forest method. In addition, a complete pipeline can optionally be used to classify the viral genus. The Fangorn Forest tool is freely available at http://geminivirus.org:8080/geminivirusdw/discoveryGeminivirus.jsp.

The geminivirus.org warehouse is structured to accommodate information about geminiviruses and related DNA satellites that become available regularly. Our platform will be frequently updated with new information extracted from GenBank, scientific publications, meetings, and abstracts. The inclusion of new data sources will enhance the wealth of data contained in our data warehouse and will promote an expansion of our system to accommodate further information that can assist in the interpretation of bioinformatics analysis results. Future improvements will permit further development of meta-analysis tools and natural language processing to extract knowledge from published studies and standardize sequences to be deposited directly into the data warehouse. We plan to develop a mobile application to assist data collection and information exchange among researchers and geminivirus.org.

## Conclusions

The geminivirus.org database is an integrated and open-access data warehouse that optimizes complicated and comprehensive searches that are difficult to perform using currently existing tools. Therefore, it is efficient in assisting targeted searches and provides accurate and concise information on all geminiviruses and geminivirus-associated satellites to the scientific community. It provides a user-friendly environment to retrieve information about (i) the geographic distribution of geminiviruses throughout the globe through an interactive map; (ii) the circular genomic structure through interactive visualization; (iii) advanced graphs with statistical information and results provided by species demarcation, phylogenetic analysis, and ORF search. Its flexibility enables the addition or analysis of various taxonomy types, genome, sampling, or biological data to facilitate and update information sources. Furthermore, the implementation of algorithms based on ML approaches allows the prediction and classification of viral genes as well as the identification of the genus based on viral genomic sequences. The data sources and additional analytical tools will greatly facilitate searches in the geminivirus.org information management system. The geminivirus.org data warehouse is freely available and will represent a valuable resource for the research community.

## Availability and requirements

geminivirus.org is available from http://geminivirus.org. The application was built in Debian Linux, GlassFish Server Open Source Edition 4.1.1, Java v8, JavaServer Pages (JSP), and MySQL5 environment. The geminivirus.org front-end layer uses HTML5, Bootstrap CSS library, JavaScript and jQuery. It is compatible with Chrome v57, Firefox v52, and Safari v10. The free tools used by geminivirus.org are: R v3.1.1, blastp/blastn v2.2.29, CLUSTAL v2.1, MAFFT v7.205, MUSCLE v3.8.31 and WEKA v3.7.11. geminivirus.org is free for academic use.

## Additional file

Additional file 1: This file includes: **Table S1.** Minimum and maximum sizes of genome sequences in each genus. **Table S2.** Number of instances/sequences of each genus contained in the dataset. **Table S3.** Performance of the genus classification model. **Table S4.** Performance of the ORF classification model. **Equations S1.** Model assessment measures. **Figure S1.** The structure of the SQL tables. (DOC 216 kb)

## Abbreviations

CDS: Coding DNA sequence
CP: Capsid protein
ETL: Extract, Transform, Load
KDD: Knowledge Discovery in Databases
ML: Machine learning
MLP: Multilayer Perceptron
MP: Movement protein
MVC: Model View Controller
NNIs: Nearest-neighbor interchange
NSP: Nuclear shuttle protein
OG: Object Geminivirus
ORF: Opening reading frame
RCA: Rolling-circle amplification
Reg: Regulatory gene
REn: Replication enhancer
Rep: Replication-associated protein
RF: Random Forest
Sd/p.sd: Symptom determinant/possible symptom determinant proteins
SDT: Sequence Demarcation Tool
SMO: Sequential Minimal Optimization
ssDNA: Single-stranded DNA
TrAP: Transactivator protein
UTM: Universal Transverse Mercator
