# Supplementary material for: Geminivirus data warehouse: a database enriched with machine learning approaches
Source: BMC Bioinformatics. 2017 May 5;18:240. doi: 10.1186/s12859-017-1646-4 (PMC5420152; doi:10.1186/s12859-017-1646-4)
Supplement: Additional file 1: — This file includes: Table S1. Minimum and maximum sizes of genome sequences in each genus. Table S2. Number of instances/sequences of each genus contained in the dataset. Table S3. Performance of the genus classification model. Table S4. Performance of the ORF classification model. Equations S1. Model assessment measures. Figure S1. The structure of the SQL tables. (DOC 216 kb) [file 12859_2017_1646_MOESM1_ESM.doc]

**Additional file 1**

**Geminivirus Data Warehouse: A database enriched with machine learning approaches**

Jose Cleydson F Silva^1,2^, Thales F M Carvalho^1^, Marcos F Basso^2^, Michihito Deguchi^2^, Welison A Pereira^2^, Roberto R Sobrinho^2^, Pedro M P Vidigal^3^, Otávio J B Brustolini^2,4^, Fabyano F Silva^5^, Maximiller Dal-Bianco^2, 4^, Renildes L F Fontes^6^, Anésia A Santos^2,7^, Francisco Murilo Zerbini^2,8^, Fabio R Cerqueira^1&^ and Elizabeth P B Fontes^2,4& *^

^1^ Departamento de Informática, Universidade Federal de Viçosa, Viçosa, Brazil.

^2^ National Institute of Science and Technology in Plant-Pest Interactions/BIOAGRO, Universidade Federal de Viçosa, Viçosa, Brazil.

^3^ Núcleo de Biomoléculas, Universidade Federal de Viçosa, Viçosa, MG, Brazil.

^4^ Departamento de Bioquímica e Biologia Molecular, Universidade Federal de Viçosa, Viçosa, Brazil.

^5^ Departamento de Zootecnia, Universidade Federal de Viçosa, Viçosa, Brazil.

^6^ Departamento de Solos, Universidade Federal de Viçosa, Viçosa, Brazil.

^7^ Departamento de Biologia Geral, Universidade Federal de Viçosa, Viçosa, Brazil.

^8^ Departamento de Fitopatologia, Universidade Federal de Viçosa, Viçosa, Brazil.

^*^ Corresponding author: bbfontes@ufv.br

^&^ Contributed equally

**Contents**

Supplementary Tables [2](#__RefHeading___Toc351103691)

Table S1. Minimum and maximum sizes of genome sequences in each genus. [2](#__RefHeading___Toc351103692)

Table S2. Number of instances/sequences of each genus contained in the dataset. [3](#__RefHeading___Toc351103693)

Table S3. Performance of the genus classification model. [4](#__RefHeading___Toc351103694)

Table S4. Performance of the ORF classification model. [5](#__RefHeading___Toc351103695)

Supplementary Equations [6](#__RefHeading___Toc351103696)

Equations S1. Model assessment measures. [6](#__RefHeading___Toc351103697)

Supplementary Figures [7](#__RefHeading___Toc351103698)

Figure S1. The structure of the SQL tables. [7](#__RefHeading___Toc351103699)

# **Supplementary Tables**

## Table S1. Minimum and maximum sizes of genome sequences in each genus.

| Genus | Minimum size | Maximum size |
| --- | --- | --- |
| Begomovirus | 2,411 | 2,959 |
| Mastrevirus | 2,425 | 2,982 |
| Eragrovirus | 2,845 | 2,854 |
| Turncurtovirus | 3,044 | 3,081 |
| Curtovirus | 3,011 | 3,180 |
| Topocuvirus | 2,961 | 2,961 |
| Becurtovirus | 2,939 | 2,960 |
| Capulavirus | 2,550 | 2872 |
| Grablovirus | 3,105 | 3,205 |
| Unclassified | 2,483 | 3,308 |
| Betasatellites | 731 | 1,552 |
| Alphasatellites | 955 | 1,579 |

## Table S2. Number of instances/sequences of each genus contained in the dataset.

| **Dataset** | **Class** | **Amount of instances** |
| --- | --- | --- |
| Genus training set | Betasatellites | 258 |
|  | alphasatellites | 302 |
|  | Becurtovirus | 23 |
|  | Curtoviruses | 18 |
|  | Eragroviruses | 4 |
|  | Mastreviruses | 57 |
|  | Topocuviruses | 1 |
|  | Turncurtoviruses | 20 |
|  | Begomovirus (DNA-A /DNA-B) | 602 |
|  | Capulaviruses | 17 |
|  | Grabloviruses | 31 |
|  | **Total** | **1333** |
| Genus test set | Betasatellites | 986 |
|  | alphasatellites | 303 |
|  | Becurtovirus | 10 |
|  | Curtoviruses | 95 |
|  | Eragroviruses | 2 |
|  | Mastreviruses | 1524 |
|  | Topocuviruses | 0 |
|  | Turncurtoviruses | 27 |
|  | Begomovirus (DNA-A /DNA-B) | 6216 |
|  | Capulaviruses | 9 |
|  | Grabloviruses | 16 |
|  | **Total** | **9188** |
| ORF training set | betaC1 | 251 |
|  | alphaRep | 240 |
|  | Rep | 631 |
|  | TrAP | 531 |
|  | REn | 525 |
|  | sd/p.sd | 447 |
|  | AC5 | 54 |
|  | CP | 644 |
|  | pre-coat | 421 |
|  | Reg | 84 |
|  | MP | 101 |
|  | NSP | 106 |
|  | **Total** | **4035** |
| ORF test set | betaC1 | 872 |
|  | alphaRep | 249 |
|  | Rep | 6435 |
|  | TrAP | 4428 |
|  | REn | 4491 |
|  | sd/p.sd | 4377 |
|  | AC5 | 724 |
|  | CP | 6330 |
|  | pre-coat | 3840 |
|  | Reg | 171 |
|  | MP | 1002 |
|  | NSP | 999 |
|  | **Total** | **33918** |

## Table S3. Performance of the genus classification model.

| **Type of evaluation** | **ML algorithm** | **Weighted average among all classes** | | | | |
| --- | --- | --- | --- | --- | --- | --- |
|  |  | **Accuracy** | **Precision** | **Recall** | **F-Measure** | **AUC** |
| Use of a test set | Multilayer Perceptron | 0.941 | 0.963 | 0.941 | 0.951 | 0.971 |
|  | SMO | 0.835 | 0.865 | 0.835 | 0.795 | 0.816 |
|  | Random Forest | 0.934 | 0.941 | 0.934 | 0.936 | 0.988 |
| 10-fold cross validation | Multilayer Perceptron | 0.970 | 0.970 | 0.971 | 0.970 | 0.991 |
|  | SMO | 0.920 | 0.901 | 0.920 | 0.906 | 0.962 |
|  | Random Forest | 0.966 | 0.966 | 0.966 | 0.965 | 0.997 |
| Leave-one-out | Multilayer Perceptron | 0.987 | 0.988 | 0.988 | 0.988 | 0.995 |
|  | SMO | 0.944 | 0.938 | 0.945 | 0.939 | 0.946 |
|  | Random Forest | 0.991 | 0.991 | 0.991 | 0.991 | 0.999 |
| Mean performance | Multilayer Perceptron | 0,966 | 0,974 | 0,967 | 0,970 | 0,986 |
|  | SMO | 0,900 | 0,901 | 0,900 | 0,880 | 0,908 |
|  | Random Forest | 0,964 | 0,966 | 0,964 | 0,964 | 0,995 |

## Table S4. Performance of the ORF classification model.

| **Type of evaluation** | **ML algorithm** | **Weighted average among all classes** | | | | |
| --- | --- | --- | --- | --- | --- | --- |
|  |  | **Accuracy** | **Precision** | **Recall** | **F-Measure** | **AUC** |
| Use of a test set | Multilayer Perceptron | 0.972 | 0.973 | 0.973 | 0.972 | 0.985 |
|  | SMO | 0.976 | 0.977 | 0.976 | 0.976 | 0.995 |
|  | Random Forest | 0.981 | 0.982 | 0.982 | 0.982 | 0.998 |
| 10-fold cross validation | Multilayer Perceptron | 0.970 | 0.971 | 0.971 | 0.971 | 0.994 |
|  | SMO | 0.972 | 0.973 | 0.973 | 0.973 | 0.994 |
|  | Random Forest | 0.976 | 0.977 | 0.977 | 0.977 | 0.997 |
| Leave-one-out | Multilayer Perceptron | 0.983 | 0.984 | 0.983 | 0.983 | 0.994 |
|  | SMO | 0.982 | 0.983 | 0.982 | 0.982 | 0.996 |
|  | Random Forest | 0.990 | 0.991 | 0.991 | 0.991 | 0.999 |
| Mean performance | Multilayer Perceptron | 0,975 | 0,976 | 0,976 | 0,975 | 0,991 |
|  | SMO | 0,977 | 0,978 | 0,977 | 0,977 | 0,995 |
|  | Random Forest | 0,982 | 0,983 | 0,983 | 0,983 | 0,998 |

# **Supplementary Equations**

## **Equations S1. Model assessment measures.**

| 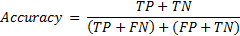 |
| --- |
| 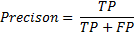 |
| 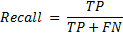 |
| 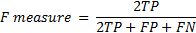 |

TP: no. true positives

TN: no. true negatives

FP: no. false positives

FN: no. false negatives

# **Supplementary Figures**

## **Figure S1**

**
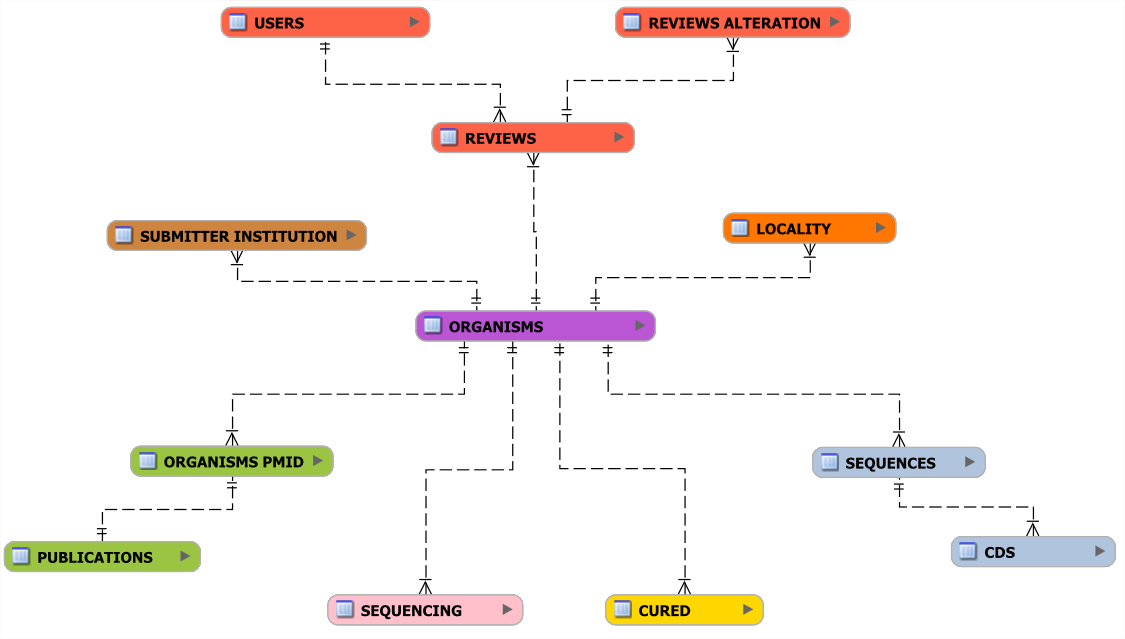
**

**Figure S1. The structure of the SQL tables.** The entity model and high-level relationship display the relational structure of the SQL tables. The fact table of the star scheme is indicated in purple. This table stores information related to the isolated (collected samples) as well as the name of species. The other tables represent the dimension tables. The red tables store information referring to the action of the users (data curation, updating, and standardization). The orange table refers to the geo-referred data. The blue tables store molecular data (genomic sequence and proteins). In yellow, the information about data curation is shown. The pink table denotes the sequencing method. In green, the scientific publications related to geminiviruses are indicated and the brown table refers to the institutions and authors associated with the full-length genomic sequences.
